# Carcinogenicity of Aspartame: Soffritti Responds

**DOI:** 10.1289/ehp.10881R

**Published:** 2008-06

**Authors:** Morando Soffritti

**Affiliations:** European Foundation of Oncology and Environmental Sciences “B. Ramazzini”, Cesare Maltoni Cancer Research Center, Bologna, Italy, E-mail: crcfr@ramazzini.it

Magnuson and Williams’s letter is substantially a repetition of the arguments set forth in a recent article (Magnuson et al. 2007), which was a “safety evaluation” sponsored entirely by Ajinomoto, the manufacturer of aspartame. Their article (Magnuson et al. 2007) and this letter contain numerous erroneous statements about the long-term carcinogenesis studies on aspartame conducted by the European Ramazzini Foundation (ERF).

First, Magnuson and Williams imply that our findings (Soffritti et al. 2007) should be discounted because the incidence of lymphomas/leukemias in the high-dose group “were within or near the reported historical control ranges.” As reported in our study (Soffritti et al. 2007), the incidence of lymphomas/leukemias observed in both sexes treated with 2,000 ppm aspartame is nearly double the concurrent control (Soffritti et al. 2007). The suggestion that concurrent control data should be ignored is contrary to the widely accepted standard of good laboratory science.

Second, Magnuson and Williams attribute our findings (Soffritti et al. 2007) to some kind of bias (i.e., infection) that would affect only treated animals but not the controls. We have responded in detail to this hypothesis in our article (Soffritti et al. 2007) and in an earlier letter (Soffritti 2006). To support their assertion, Magnuson and Williams mislead readers by stating that “the lung was often the site of lymphoma again in this [second] study.” However, we actually reported that

we observed the diffusion of neoplastic tissue not only in the lung but also concurrently in various organs (liver, spleen, mediastinal and other lymph nodes). (Soffritti et al. 2007)

Infection as a mode of action for induction of rat lymphoma has been recently examined by a group of scientists at the National Center for Environmental Assessment of the U.S. Environmental Protection Agency; Caldwell et al. (2008) found that

a careful examination of available information does not support the hypothesis that the observed lymphomas/leukemias in the ERF bioassays are a general effect from infection. The reports of chemically-induced lymphomas/leukemias by the ERF seem to be chemical specific.

Third, the idea that we must provide a “biologically plausible explanation” for human or rodent carcinogens is a time-honored approach to postpone or prevent the application of regulatory measures to minimize carcinogenic risks. The reality is that this explanation is quite often unknown, as is, in general, the mode of action behind the carcinogenic process.

I regard the other questions raised by Magnuson and Williams as trivial. For example, whatever the doses at various ages and weights, the finding of any effect should be a cause for concern. Likewise, the authors’ observation that some methodologic details were omitted from the publication certainly does not change the oncologic results of this research.

Magnuson and Williams express disappointment that *Environmental Health Perspectives* would publish original scientific research by the ERF after regulatory agencies went through so much trouble to review our first aspartame study (Soffritti 2006) only to disagree with our conclusions. It is the obligation of the agencies responsible for food safety to review any new scientific data available and to make their opinion available to the public. The Food and Drug Administration (FDA) did not make public the contents of their review, but rather they issued a short press release a full year after the European Food Safety Authority (EFSA) concluded its evaluation, and coincidently, just days before I presented new aspartame data in a lecture at the Mount Sinai School of Medicine in New York (FDA 2007).

I find it unfortunate that some scientists have such a low tolerance for original, independent scientific research; however, I welcome continued discussion and more importantly, additional long-term experimental studies on aspartame and other artificial sweeteners. We at the ERF stand behind our results, and we remain convinced that a review of the current regulations governing the use of aspartame is necessary to better protect public health.
